# Comprehensive Review on Application Progress of Marine Collagen Cross-Linking Modification in Bone Repairs

**DOI:** 10.3390/md23040151

**Published:** 2025-03-30

**Authors:** Xiaofei Zhai, Xinrong Geng, Wenjun Li, Hongli Cui, Yunqing Wang, Song Qin

**Affiliations:** 1 Research Institute of Marine Traditional Chinese Medicine (Qingdao Academy of Chinese Medical Sciences), Shandong University of Traditional Chinese Medicine, Qingdao 266112, China; xiaofeiz128@163.com (X.Z.); xinronggeng99@163.com (X.G.); wjli@yic.ac.cn (W.L.); sqin@yic.ac.cn (S.Q.); 2 Yantai Institute of Coastal Zone Research, Chinese Academy of Sciences, Yantai 264003, China

**Keywords:** marine collagen, cross-linking modification, bone repair, application

## Abstract

Bone tissue injuries are a significant health risk, and their repair is challenging. While various materials have potential for bone repair, issues like sourcing and immune rejection limit their use. Marine-derived collagen, abundant and free from religious and disease transmission concerns, is a promising biomaterial in bone tissue engineering. Cross-linking modification can enhance its mechanical properties and degradation rate, making it more suitable for bone repair. However, detailed analysis of cross-linking methods, property changes post-cross-linking, and their impact on bone repair is needed. This review examines marine collagen’s modification methods, improved characteristics, and potential in bone tissue repair, providing a foundation for its effective use in bone tissue engineering.

## 1. Introduction

Bones are vital for body structure and function, providing support and protection during participation in daily activities. Despite their strength, bones are susceptible to damage from aging, stress, and illness, emphasizing the importance of maintaining bone health [1]. The complex process of bone healing aims to restore bone tissue integrity and preserve health. In modern surgery, bone repair poses significant challenges. Common methods include autografts, allografts, and artificial bone grafts, each with limitations [2]. Autografts are limited by donor site scarcity and potential for injury, while allografts and artificial materials carry risks of viral infection and immune rejection, constraining their clinical use [3].

The orthopedic field of bone repair has thus made it imperative to find more appropriate materials for bone repair, and the ideal materials must meet the following requirements: mechanical tolerance, biodegradability, biocompatibility, and bone-induced regeneration [2]. Apart from the restricted availability of autologous bone, conventional bone repair materials typically cause immunological rejection and exhibit low biological activity [4]. For this reason, finding better repair materials has never been an easy task in terms of bone tissue restoration.

Collagen, with its excellent biological properties, has become a promising biomaterial in various medical fields such as hemostasis, orthopedics, and oral health. It is a key component of the extracellular matrix (ECM), playing a vital role in tissue repair and ECM integrity [5]. As the largest treasure house of resources on the earth, the ocean contains rich sources of collagen, such as fish skin, scales and bones of fish, as well as invertebrates such as sea cucumbers, jellyfish, and sponges [6]. Different extraction techniques, including enzymatic hydrolysis, acid and alkali extraction, as well as ultrasound-assisted extraction, are specifically designed to target distinct types of collagen and can effectively maximize the utilization of resources [7]. Marine collagen (MC), which is abundant and free from religious restrictions and disease risks, is particularly significant in bone tissue engineering (BTE) due to its bone conductivity, biocompatibility, and cell adhesion [8]. Collagen’s triple-helix structure provides mechanical integrity and structural support for organs of higher animals [9]. In bone injury, collagen-based scaffolds can replace damaged tissue, promoting cellular activity and new bone integration with the host tissue. Marine collagen is essential for the ECM, as it inhibits inflammation, induces chondrocyte differentiation, increases bone mineral density, and promotes osteogenesis and collagen synthesis [4]. However, pure type I collagen materials are unstable and degrade quickly, limiting their effectiveness in bone replacement and clinical use [10]. To enhance the application of MC in bone tissue regeneration, it is crucial to modify collagen to improve its mechanical strength and stability and reduce biodegradation by forming intermolecular bonds. This modification is essential for increasing the widespread application of MC in bone tissue engineering [11].

**Figure 1:**
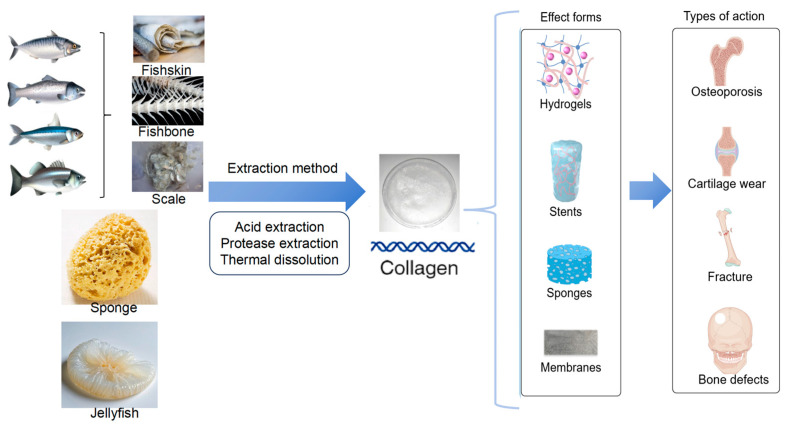
Source, extraction, application form, and action type of marine collagen.

Considering the properties of MC and the current state of research on collagen modification methods, this paper addresses the properties of MC used in bone repair, and the modified MC used in various forms of bone repair (Figure 1).

## 2. Introduction of Marine Collagen

### 2.1. Characteristics and Physicochemical Properties of Marine Collagen

Occupying more than 70% of the global surface area, the oceans and seas are the largest ecosystems on the planet, harboring a wealth of species resources and offering broad prospects for development. MC may be extracted from a range of aquatic creatures, such as algae, vertebrates, and invertebrates [12]. Collagen made from fish skin helps minimize the pollution caused by a large amount of waste, and this action also increases the value of marine byproducts [13]. MCs are widely utilized in medicine, cosmetology, food, health care, and other sectors. Their distinctive benefits include high yield and low production cost due to their superior biological compatibility, high biodegradability, low immunogenicity, good water solubility, high safety, and easy extraction.

Marine collagen, especially collagen from marine organisms such as fish, has a certain degree of similarity in amino acid sequence and structure with human collagen, and usually has low immunogenicity and relatively low probability of causing allergic reactions [14]. Land-derived collagen and related products derived from livestock such as cattle and pigs may cause bovine spongiform encephalopathy [7]. Of course, individuals with shellfish allergies may also have adverse reactions to marine-sourced collagen [15]. Therefore, the specific allergy needs to be judged according to the individual constitution, product quality, processing technology, and other factors.

To date, the internal structure of MCs has been shown to be intricate, and approximately 20 distinct collagen proteins have been identified. Among these, type I collagen is the most prevalent. Type I collagen is characterized by a four-level structural organization, which encompasses fibril structures, α-helices, triple helices, and amino acid triplets. The type I collagen trimer is composed of three polypeptide α-chains that adopt a characteristic triple-helix conformation. This protein is rich in specific amino acid residues, such as hydroxyproline, proline, and glycine [16]. The heat resistance of type I collagen is significantly influenced by the degree of hydroxylation of proline and lysine; specifically, a higher quantity of amino acids corresponds to greater heat resistance [17].

The yields and physicochemical properties of collagen derived from marine species are highly dependent on the extraction method employed Acid solubilization extractionand pepsin-mediated solubilization extraction are two commonly used techniques. Multiple studies have demonstrated that combining the acid solubilization method with pepsin treatment can effectively enhance the yield of collagen extraction [18].

MC is a high-grade biocompatible substance that creates the perfect conditions for cell attachment and proliferation. The MC structure is rich in amino acids and highly hydrophilic and can generate an appropriate milieu for cell development by interacting with the ECM. In the field of the ECM, MCs are widely used to rejuvenate and repair bone tissue. They can stimulate osteoblast-differentiated MSCs, encourage osteoblast proliferation, and preserve the immunomodulatory functions of osteoblasts [19]. Overall, the use of MC is a safe, practical, and promising solution for the ECM.

### 2.2. Modification of Marine Collagen

**Figure 2:**
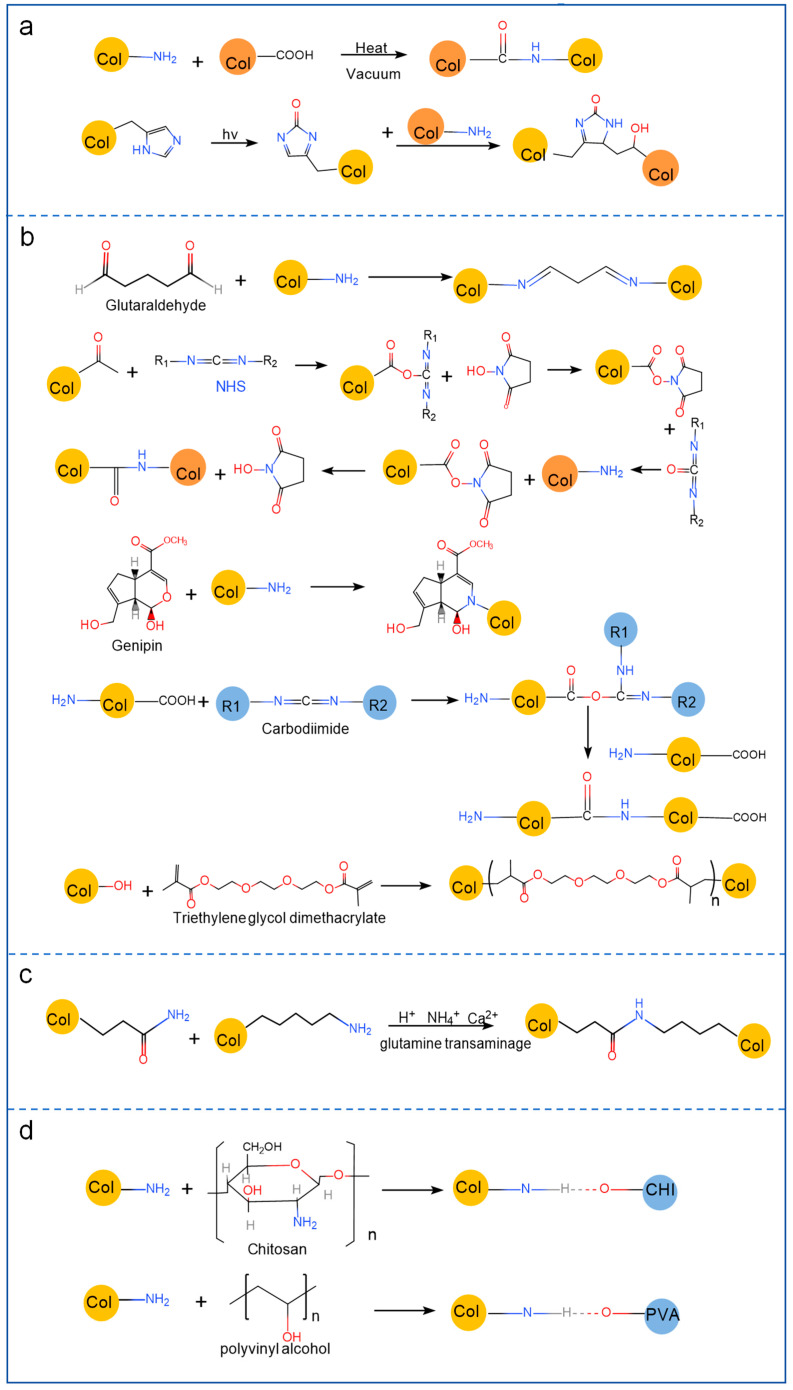
Modification methods of collagen. (**a**) Physical modification. (**b**) Chemical modification. (**c**) Enzymatic modification. (**d**) Polymer material blending modification.

When collagen is used alone, defects such as an unstable degradation rate, poor mechanical properties, and poor water resistance can occur [20]. Collagen must thus be changed to satisfy the evolving demands of the bone tissue regeneration sector. Currently available collagen modification techniques include physical modification, chemical modification, enzymatic modification, and mixed polymer material blending modification. Below is a description of the research status of various alteration approaches. Figure 2 shows four modification methods for collagen.

#### 2.2.1. Physical Modification

Physical modification is the process of altering the surface of collagen using extreme heat, dehydration, UV light, and γ-ray radiation without the use of chemicals. The goal is to enhance the overall functionality of collagen. The physical cross-linking method of modifying collagen offers several benefits, including low cost, gentle reaction conditions, and the prevention of hazardous chemicals in collagen tissue. However, due to its low resistance to collagenase, it tends to cause degradation and denaturation of collagen easily. Moreover, its cross-linking strength and stability are not high, which restricts its practical application in collagen modification [11]. At low γ radiation doses (10 and 20 kGy), cross-linking of peptide chains predominates, resulting in tighter packing, lower chain mobility, and higher thermal stability [21]. UV-mediated cross-linking preserves native-like cell binding, proliferation, and surface colonization, allowing dose-dependent expansion of the stability of collagen-based materials in aqueous environments [22]. Chen et al. [23] exposed collagen membranes to extreme dehydration heat (DHT) and found that the degree of degradation increased with increasing treatment temperature and duration. Additionally, they observed that the DHT treatment enhanced the tensile strength and cross-linking density, and this effect was related to the treatment temperature.

#### 2.2.2. Chemical Modification

A variety of active groups, including amino, carboxyl, and hydroxyl groups, are present in collagen molecules. Chemical modification is essentially the introduction of functional groups into collagen that confer better mechanical properties by chemically reacting with the active functional groups in collagen. Chemical modification is the most popular modification technique and may fall into one of three groups: chemical cross-linking modification [11], side chain group modification [24], and graft copolymer modification [25].

To achieve this modification goal, chemical cross-linking mainly involves reacting the material with the active group of collagen. Glutaraldehyde (GA), genipine, N-hydroxy succinimide (NHS), and 1-ethyl-3-(3-dimethylaminopropyl) carbodiimide (EDC) are examples of chemical cross-linkers that are frequently utilized [26]. Zhang et al. [27] modified and cross-linked collagen films using GA as a cross-linking substance. According to the study, the tensile strength, fracture elongation, and water absorption of collagen films all dramatically increased following cross-linking. This finding suggested that adding GA could greatly enhance the mechanical and swelling properties of collagen films. Lin et al. [28] used EDC and NHS to catalyze the formation of a composite hydrogel scaffold with a network structure of carboxymethyl chitosan and collagen, which performed well in biocompatibility tests. This method can promote cell adhesion and proliferation, achieve optimal mechanical properties and microstructure of the hydrogels, and improve the biocompatibility of scaffolds.

Side chain group modification involves the addition of active groups, such as hydroxyl and carboxyl groups, to the side chain of collagen to alter its charge distribution and provide new characteristics. For instance, amination agents, such as ethylenediamine, diethylenetriamine, and poly ethylenimine, are commonly used to modify the aminination of collagen [24].

The process of attaching a new polymer chain to the structure of an existing polymer using radical polymerization, ring-opening polymerization, or addition polymerization is known as graft copolymer modification, and currently, ene monomers such as butyl acrylate, methyl propionate, and acrylonitrile are the most commonly used copolymers [25]. Semenycheva et al. [29] reported that the grafted copolymer of collagen and acrylate improved the solubility of the grafted copolymer and facilitated the release of water during the vacuum drying process, leading to the production of a polymer composite membrane. Chemical cross-linking has a better cross-linking effect and greater resistance to collagenase than physical cross-linking, but it is also more expensive and has higher cytotoxicity overall.

#### 2.2.3. Enzymatic Modification

High catalytic efficiency, mild reaction conditions, and preservation of the triple-helix structure are characteristics of enzymatic modification. Glutaminase and polyphenol oxidase are two frequently utilized enzymes [30]. Although there are no chemical residues with this approach, it is not often utilized for modification research because it is challenging to control the cross-linking reaction. Sommer [31] found that when using transglutaminase to cross-link collagen from silver carp skin and combine it with bacterial cellulose in the preparation of composites, the cross-linking density, thermal stability, and tensile strength were increased, resulting in better mechanical properties. Enzyme-mediated collagen membranes can lead to significant enhancement of biomechanical properties while maintaining their structural integrity [32]. The mesh of the hydrogel is closely related to the enzyme size, and the degradation rate decreases with the initial cross-linking density of the hydrogel [33].

#### 2.2.4. Polymer Material Blending Modification

Beyond the methods previously described, other polymer molecules can be utilized to modify collagen. In theory, blending modifications can be classified into two main types: physical blending, which encompasses solution blending, mechanical blending, and emulsion blending, and chemical blending. Collagen, as a polymeric compound, is capable of undergoing chemical blending with other polymers. Through this process, polymers with electrostatic or hydrogen-bonding effects can be formed, thereby enhancing the spinnability of collagen. The primary types of polymer materials that interact with collagen include both natural and synthetic varieties. Among these, natural polymers are extensively used for collagen blending. Their popularity stems from several advantageous characteristics, such as easy accessibility, high biocompatibility, and biodegradability. The most common natural polymers employed in this context are chitosan, cellulose, and cellulose derivatives [34]. In the case of collagen–chitosan composites formed by the combination of collagen and chitosan, cross-links are established between the amino, hydroxyl, and other functional groups of the two components. Under external conditions, collagen–chitosan complexes are formed via electrostatic interactions and hydrogen bonds, resulting in the creation of composites with three-dimensional structures. Zhanget al. [35] conducted an investigation into the characteristics of films composed of bone collagen–chitosan combinations with varying mixing ratios using two-dimensional infrared correlation spectroscopy. The results of their study demonstrated that, in comparison to pure single-component polymer films, the combination of chitosan and collagen molecules led to the formation of a more stable and superior film structure. Regarding synthetic polymeric materials, biodegradable polymers like polylactic acid, polyvinyl alcohol, and polyglycolic acid, as well as non-biodegradable polymers such as polyurethane, polyamide, and polymethacrylate, can be combined with collagen [36]. For example, the abundant hydroxyl groups present in polyvinyl alcohol can form hydrogen bonds with the amino groups of collagen, enabling the creation of a more stable macromolecular material. Lang et al. [37] copolymerized modified collagen polypeptides with waterborne polyurethane to prepare a solvent-free WPU-g-C copolymer emulsion and found that its storage modulus can reach 7000 MPa, and its mechanical properties are superior, which is more conducive to its application as a bone healing material.

## 3. Property Characterization of Collagen Application in Bone Repair

**Figure 3:**
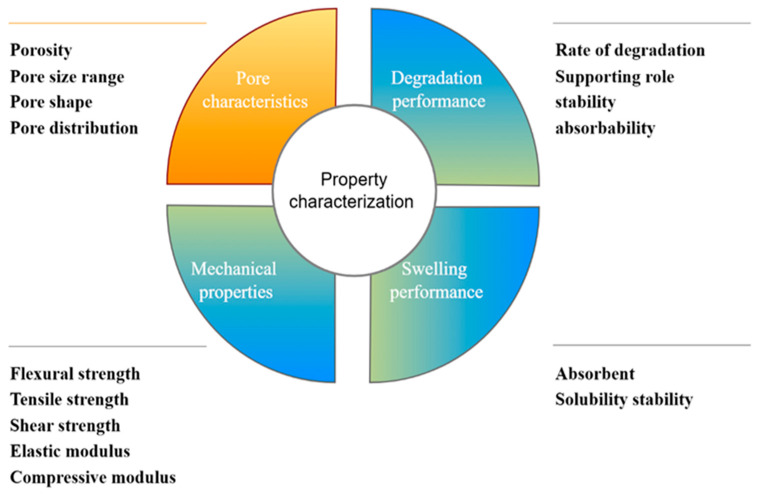
Study of the application properties of collagen in bone repair.

MC has emerged as a significant bioactive material with extensive applications in the realm of tissue engineering. However, one of the major limitations of collagen, including MC, is its lack of sufficient mechanical properties for in vitro therapy or cell culture. In many tissue engineering applications, especially those requiring load-bearing capabilities, the inherent mechanical weakness of collagen can pose challenges. For instance, in bone tissue engineering, the scaffold needs to withstand mechanical stress during the process of bone regeneration and remodeling [38]. The fundamental properties of collagen, such as its mechanical strength, stability, and degradation rate, are significantly influenced by processing and cross-linking techniques. Different processing methods can lead to variations in the final structure and properties of the collagen-based materials (Figure 3).

### 3.1. Pore Characteristics

Pore characteristics constitute one of the crucial parameters of marine-collagen-based materials in the field of bone repair. These characteristics, including pore size, shape, distribution mode, and porosity, have a profound impact on the process of bone tissue repair [39]. Pore size is a crucial factor with diverse requirements depending on different parts and forms of materials. Currently, the pore sizes of commonly used bone repair materials generally range from 100 to 400 μm, while the range for optimal bone conduction is 200–350 μm [40]. In clinical practice, the pore sizes of stent materials typically fall within 200–350 μm. This size range enables efficient waste removal from the body and promotes cell penetration, the formation of the ECM, and the transfer of nutrients [41]. As Hulbert et al. [42] pointed out, different pore sizes can lead to distinct tissue growth scenarios. Some bone tissue can grow in relatively large pores of 100–200 μm. As the pore size decreases, unmineralized bone-like tissue can grow, and when the pore size further reduces to 10–75 μm, only fibrous tissue can pass through. The minimum pore diameter for a bone tissue regeneration material is usually 100 μm. When MC serves as the matrix for a scaffold, the minimum pore diameter is commonly 52 μm. On the other hand, implant materials for areas with bone defects usually demand osteogenic pores larger than 300 μm. Freeze-drying was used by Grabska-Zielinska et al. [43] to create a three-dimensional scaffold made of dialdehyde starch cross-linked with silk fibroin, collagen, and chitosan. This scaffold had a porosity of approximately 90%, and the connection pore size was smaller than 200 μm. By adding cross-linkers, the physical properties of collagen-based materials can be altered. Currently, collagen-based materials for bone tissue formation typically have porosities greater than 90%. This high level of porosity guarantees sufficient nutrient exchange and provides a favorable environment for cell multiplication, which is essential for the successful repair and regeneration of bone tissue [44]. The pore shape and distribution mode also interact with cell behavior and tissue ingrowth, but more in-depth research is needed to fully understand their complex relationships in the context of MC-based bone repair materials.

### 3.2. Degradation Performance

One of the essential characteristics of bone repair materials is their degradability. This property enables them to degrade within the body and be gradually replaced by autologous bone. During the degradation process, they can serve as a support structure, providing a temporary framework for the growth and development of new bone tissue. Maintaining the integrity of the bone–stent system and promoting new bone formation throughout the healing stage hinges on the ability of the stent material to break down at an appropriate rate [45]. MC can improve pore regularity and the stability of water conditions by changing cross-linking. According to Blackstone et al. [46], the multifunctional structure and biocompatibility of collagen following electrospinning treatment make it the perfect material for scaffolds. However, the material is prone to structural changes during preparation, which increases its degradation rate and makes it difficult to meet the requirements of many fields. Chemical cross-linking of collagen using electrostatic spinning technology can efficiently enhance the stability of the material and significantly improve cell metabolism in vitro. In addition, Pripatnanont [47] demonstrated that semi-absorbable barrier membranes made from a combination of silk fibroin, glycerol, and fish gelatin as the raw materials have a lower degradation rate compared to the materials used individually. Jinet al. [48] fabricated a poly (lactic-co-glycolic acid) nanofiber membrane composed of nanohydroxyapatite and collagen. This membrane exhibited low immunogenicity. Moreover, through electrospinning, it significantly improved the degradation properties of the fiber and guided bone regeneration (GBR). This membrane may direct bone regeneration through the electrospinning process, which can create a suitable microenvironment for cell adhesion and growth. After cross-linking, the degradability of marine-collagen-based materials greatly improves, and this enhanced degradability makes MC more suitable for use in the field of skeletal repair.

### 3.3. Mechanical Properties

MC has emerged as a promising polymer material in tissue engineering, primarily due to its outstanding cell affinity and remarkable biocompatibility. These properties enable MC to interact favorably with cells, promoting cell adhesion, proliferation, and differentiation, which are essential processes in tissue regeneration. However, despite its many advantages, the mechanical properties of MC still need enhancement to fully meet the demands of various tissue engineering applications, especially those related to bone repair. The mechanical characteristics of bone tissue repair materials play a pivotal role in bone regeneration and repair. They are crucial for maintaining the stable structure of biomaterials, emulating the microstructure of the ECM, and regulating cell-to-cell interactions, as well as the migration and distribution of nutrients and metabolites within tissues. All these factors are essential for controlling cell growth and function [49]. Natural tissues exhibit a wide range of mechanical properties, which results from differences in tissue types, internal structures, and compositions. When considering materials for bone replacement, it is essential that their ideal mechanical properties align with those of natural bone. Among different types of bones, the femur has the largest modulus of elasticity, indicating its high resistance to deformation under tensile or compressive forces. The ulna and radius have the highest bending strength, which is crucial for withstanding bending loads. The bending modulus and bending strength also vary among different natural bones. Cancellous bone typically has a bending strength of around 20 MPa and a bending modulus of approximately 2 GPa. In contrast, dense bone has a much higher bending strength, ranging from 110 to 200 MPa, and a bending modulus between 10 and 20 GPa [50]. To address the mechanical limitations of MC, various strategies have been explored. Research has shown that cross-linking techniques, such as GA cross-linking, can be effective in improving the mechanical strength of the collagen scaffold without altering its porosity or pore size [51]. Chemical cross-linking techniques can enhance the collagen mechanical characteristics and bring the modified collagen characteristics closer to those of natural bone. An increasing body of research demonstrates that mechanical attributes such as strength and the collagen modulus can be altered for better results. The stiffness, compressive modulus, tensile strength, and ductility of MC-based biomaterials are markedly increased following cross-linking modification [52].

#### 3.3.1. Modulus

As a fundamental parameter in evaluating the mechanical properties of biomaterials for tissue engineering applications, the modulus quantifies a material’s resistance to elastic deformation under applied stress. This critical characteristic is typically characterized through various parameters, including the modulus of elasticity, shear modulus, and compression modulus [53]. It has been shown that the addition of hydroxyapatite (HAP) can more than double the modulus of collagen. Compared with those of pure collagen scaffolds, collagen scaffolds enhanced with nanosized bio-glass have demonstrated a greater compression modulus. Haugh et al. [52] reported that collagen–GAG scaffolds cross-linked with EDC or NHS showed a greater compression modulus than pure collagen–GAG scaffolds did. The compressive strength of these scaffolds increased fourfold, reaching 1.8 kPa, following treatment with 96 mM EDC. Raftery et al. [54] detected changes in the volume compression modulus when chitosan was added to collagen obtained from salmon. The compression modulus of the chitosan-free collagen scaffold was only 0.13 kPa, but as the chitosan content increased, the compression modulus increased by 4–5.5 times.

#### 3.3.2. Strength

In the field of tissue engineering materials, strength pertains to the material’s capacity to endure mechanical stresses, including metrics such as flexural strength, shear strength, yield strength, compressive strength, and tensile strength. Incorporating inorganic elements such as calcium phosphate, HAP, and bio-glass into traditional collagen production processes can enhance the strength and modulus of pure collagen scaffolds. Collagen and chitosan have molecular compatibility and can be used to create stable complexes. In the DHT cross-linking of collagen scaffolds, certain mechanical properties (such as tensile strength and compression modulus) can increase with increasing temperature. Overall, cross-linking can greatly increase the mechanical strength and modulus of collagen materials [55]. Haugh et al. [56] reported that better mechanical characteristics can be achieved by increasing the temperature and lengthening the DHT treatment period. Compared with cross-linking at 105 °C, DHT cross-linking at 180 °C resulted in at least twofold increases in compressive strength and tensile strength. The cross-linking of type I collagen–chitosan membrane with EDC, according to Hidalgo-Vicelis [57], may enhance the membrane’s tensile strength and its chemical characteristic of enzyme resistance, which offers insightful recommendations for the application strategy of GBR. Collagen, elastin, and chitosan scaffolds were cross-linked using carbodiimide by M.N. Taravel et al. [58]. Compared with the collagen–elastin scaffold, a higher percentage of chitosan (10%) could bind to the collagen scaffold, and cross-linking improved the tensile strength and modulus.

### 3.4. Swelling Performance

Another crucial component of bone healing materials is their swelling performance, which exerts a substantial influence on the utilization of these materials. The binding of collagen to different substances has a direct impact on the variation of its swelling characteristics. Xue et al. [59] reported that the treatment of heterogeneous collagen membranes with DHT remarkably enhanced their stability and compressive strength when the membranes were in a swollen state. This performance improvement holds great promise for guiding bone regeneration. Valipour [60] investigated the effect on the physicochemical properties by adding different volumes (0.5, 1.0, and 1.5 mL) of dialdehyde starch to a mixed hydrogel of fish-skin collagen and chitosan. The study revealed that the amount of dialdehyde starch significantly affected the swelling rate and biodegradability of the hydrogel. According to Zhang et al. [61], cross-linked hydrogels generally expanded more quickly than non-cross-linked polymer membranes.

## 4. Application of Marine Collagen to Restore Injured Bone Tissue

Table 1 shows the attributes of MC as well as the features of various bone tissue manufacturing techniques. MC is a new biomaterial that has the capacity to repair or regulate abraded or injured bone tissue by altering the cross-linking between collagen molecules. This biomaterial has many potential applications in the regeneration of bone tissue.

Modified collagen-based biomaterials can be fabricated into various physical forms, including hydrogels, composite scaffolds, membranes, and sponges. These forms are extensively employed in vivo for further bone matter regeneration in a range of therapeutic settings. This portion of the review discusses the study of several processed forms of modified MC for use in the tissue engineering of bones.

### 4.1. Collagen Hydrogels

Hydrogels are a class of cross-linked 3D mesh polymers. Consisting of collagen-based hydrated substances combined with various natural polymers, they have remarkable properties. Because of their outstanding water solubility, high liquid permeability, strong physical properties and biocompatibility, excellent biological action, and anti-enzymatic lysis capability, collagen hydrogels are widely used in tissue engineering, local medication administration, and other applications [88].

The structure of MC hydrogels is similar to that of the ECM, and they are also highly biocompatible. Therefore, they are considered a bone tissue repair material offering a great deal of versatility. Ochi et al. [89] used collagen hydrogels packed with autologous chondrocytes to successfully repair cartilage lesions in human knee joints. However, collagen hydrogels have certain disadvantages, including poor mechanical strength, restricted capacity to stimulate stem cell development, and rapid degradation. To solve the aforementioned problems, researchers typically combine them with other synthetic polymers or mixed polymers to make them more biocompatible and allow them a wider variety of uses in repairing bone tissue.

#### 4.1.1. Collagen Hydrogels Bound with Organic Materials

**Figure 4:**
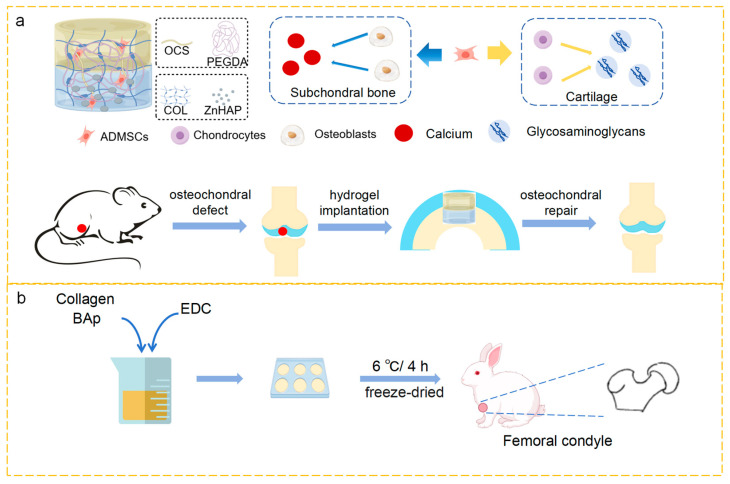
(**a**) Schematic diagram for repairing the rat model with osteochondral defect by promoting the chondrogenic differentiation and osteogenic differentiation of the bilayer hydrogel [75]. (**b**) Schematic diagram of the femoral condylar defect experiment in which a 3D composite scaffold made of blue shark skin collagen and bioapatite is employed [78].

Collagen combined with organic materials can have a synergistic effect on hydrogels. Chitosan, a natural polysaccharide, has favorable biological properties. Furthermore, chitosan can create covalent interactions with hydrogels, enhancing their stability and mechanical properties. The carbonyl and amino groups between the two biopolymers, chitosan and collagen, undergo physical interactions to function more effectively in hydrogels [90]. It has been shown that chitosan/collagen composite hydrogels enhance biocompatibility, bone conduction, and bone-forming capabilities, as well as the mechanical strength in the ECM. Azaza [71] developed a composite hydrogel made from chitosan and bluefin tuna collagen by freezing/thawing, which displayed a highly interconnected porous structure and was considered a potential tissue engineering material. Since alginate is an anionic polysaccharide with good mechanical properties, superior biocompatibility, biodegradability, and nonimmunogenicity, it has been widely applied in the field of biomedical engineering in recent years. However, the strong hydrophilicity of alginate results in limited cell adhesion and low bioavailability. Collagen binds well to cells and can be cross-linked with sodium alginate to fabricate a composite collagen hydrogel with complementary advantages and disadvantages, which is more suitable for bone tissue repair [91]. Zheng et al. [92] reported that EDC/NHS cross-linked fish scale collagen peptide-loaded calcium alginate hydrogels, which have a higher cross-linking density and higher storage modulus and loss modulus, are more suitable for bone regeneration. A porous marine hybrid structure made of fibrous jellyfish collagen and alginate stimulates hMSC chondrogenic development and has potential for articular cartilage repair. Hyaluronic acid is a nontoxic basic glycosaminoglycan that has strong biocompatibility, biodegradability, and cell affinity, and is frequently employed in the hydrogel preparation process. Fibrous gels produced by Yan et al. [93] using collagen and hyaluronic acid showed markedly enhanced performance following EDC/NHS cross-linking, suggesting potential applications in bone fracture repair. Polycaprolactone is an FDA-approved biodegradable polyester-based material that has great potential for wide clinical application due to its favorable biological and mechanical characteristics. Cao et al. [75] fabricated an injectable bilayer hydrogel that imitates the structure of natural osteochondral bone. The material was loaded with adipose-derived MSCs for the simultaneous regeneration of cartilage and enhancement of subchondral bone (Figure 4a). Hydrogel-loaded mesenchymal stem cells modified with polycaprolactone micro/nanofibers combined with collagen are effective in regenerating bone tissue [94]. Growth factors, a fundamental element of tissue engineering, are important regulators of cell growth and proliferation and directly impact bone tissue regeneration. Because platelets are rich in growth factors, they are crucial for controlling body growth and development. Previous research has indicated that PRP can accelerate the repair process of bone abnormalities by promoting early cell proliferation and differentiation [95].

#### 4.1.2. Collagen Hydrogels Bound with Inorganic Materials

HAP is a major inorganic component that makes up human bone and has biological functions that include assisting in the healing of injured bone tissue and stimulating or inducing osteogenesis. Apart from its application in biological activity and bone conduction, it is extensively incorporated into collagen-based hydrogels to enhance their rigidity. Zhang et al. [96] fabricated a synthetic bone composite comprising HAP and MC, resulting in a significant increase in the compressive strength of the composite material. Bioactive ceramics are osteoconductive, and they can form good bio-bonds with human tissue, be used as bone defect filling materials, and promote osteogenesis on the surface of scaffolds. As a result, they are widely applied in bone and dental fields. Jang et al. [97] used bioprinting technology to build a novel bio-composite hydrogel composed of nanofiber collagen and β-tricalcium phosphate, which was similar in morphology to nanofiber ECM and showed a mechanically stable structure.

### 4.2. Composite Scaffolds

Because MC scaffolds remarkably resemble the structure of real bones, they are vital for bone tissue creation. The scaffold should possess a suitable pore structure to permit the inward growth of cells and ensure their precise distribution within the porous framework. Additionally, it should have the capacity to facilitate the formation of the new structures surrounding it. The suitable pore size, porosity, and adhesion of MC scaffolds, along with their excellent mechanical qualities, can influence the regeneration of bone tissue. Fibrillated collagen from jellyfish and mineralized salmon collagen were mixed by Bernhardt et al. [79] to fabricate a biphasic scaffold that induced human mesenchymal cells to differentiate into osteogenic precursors, thereby accelerating the healing of bone and cartilage defects. Collagen materials alone are insufficient to stimulate bone tissue regeneration, as demonstrated by earlier research. As a result, scaffolds made of natural collagen cannot function optimally in vivo. Therefore, compositing these materials with other organic or inorganic materials to construct novel scaffolds can significantly enhance their osteogenic, bioactive, and mechanical qualities.

#### 4.2.1. Marine Collagen Composite Scaffolds Bound by Organic Materials

Polymers such as naturally derived polysaccharides and silk fibroins are frequently incorporated to collagen groups for the preparation of composite scaffolds. Chemical cross-linking of collagen with chitosan monomers modulates biomechanical properties to promote cell adhesion and proliferation. Elango et al. [98] employed a composite scaffold consisting of blue-shark-derived collagen and chitosan. This scaffold was able to improve the stiffness and degradation rate, effectively promoting osteoblast formation. Chondroitin sulfate, a naturally occurring sulfated glycosaminoglycan, bonds to collagen. This bonding enhances cell adhesion and decreases the degradation rate of collagen. By compounding with polycaprolactone, collagen can increase the flexibility of the cell matrix, increase cell hydrophilicity, and facilitate cell proliferation and differentiation, indicating its potential application as a scaffold material for BTE. Oh et al. [80] constructed a 3D scaffold that combines polycaprolactone with fish collagen from osteogenic abalone. They demonstrated good bone-inducing function in a rabbit model with a tibial defect, and the scaffold showed promise as a substance for tissue implantation and bone regeneration. Silk fibroin has reproducibility and desirable mechanical properties. Xue et al. [99] used silk fibroin and collagen to prepare SF/Col composite scaffolds, and thereby improved the pore structure, elastic modulus, and mechanical anisotropy; these materials are more appropriate for cartilage repair and have better applicability to cartilage defects.

#### 4.2.2. Marine Collagen Composite Scaffolds Bound by Inorganic Materials

The mechanical strength of simple collagen materials is poor. They can be made stronger, more bone-conductive, more dimensionally stable, and with a larger surface adhesion area by combination with inorganic bio-ceramics. This will enable these composite materials to be widely used as materials for bone repair. Findings demonstrated that HAP could considerably enhance the physicochemical characteristics of collagen scaffolds, increase material specific surface area, and encourage cell adhesion. The direct chemical linkages that HAP may create with the host bone facilitate the quick integration of the scaffold with the bone. After successful extraction of shark-derived collagen and HAP, Diogo et al. [81] mixed them to produce a 3D composite structure through EDC/NHS cross-linking. Its improved scaffold toughness and mechanical strength allow it to better support osteoblast adhesion and proliferation, providing a cutting-edge method for the clinical management of bone abnormalities. The 3D composite scaffolds produced by freeze-drying blue shark skin collagen with bioapatite obtained from blue shark teeth showed excellent performance in promoting bone tissue regeneration in femoral condyle defects, which were observed 12 weeks postoperatively in New Zealand rabbits [78] (Figure 4b). As a novel kind of artificial bone material with good osteoconductivity, biocompatibility, and good osteoinductive properties, β-tricalcium phosphate can be used to slow down bone degradation and improve its biological strength by changing its pore size and purity. Muthukumar [82] reported that porous composite scaffolds supplemented with HAP, chitosan, and β-tricalcium phosphate enhanced and encouraged the growth and multiplication of MG-63 cells, suggesting that the use of these porous composite scaffolds may become a viable substitute for bone grafting. Bioactive glass has high surface reactivity and good biocompatibility in both bone and soft tissue. The blending of carbon nanotubes and collagen to form a scaffold can significantly increase the stiffness of the scaffold. Three-dimensional porous composite scaffolds were created by Wu et al. [64] using genipin cross-linking technology and by freeze-drying and other techniques to process oyster shell powder and collagen derived from stingray skin. Compared with collagen alone, the use of OSP enhanced the mechanical characteristics and cellular adherence of collagen scaffolds, which could encourage cellular division and multiplication and have a substantial impact on the regeneration of bone defects.

### 4.3. Collagen Membranes

In experiments and clinical studies, various collagen membrane materials, such as polytetrafluoroethylene and collagen, have been used to achieve GBR. Collagen membranes mostly consist of type I collagen. In the middle of the 1980s, collagen membranes were initially developed as barrier membranes for regeneration. The collagen membrane is a kind of material that serves as a barrier, which is mainly used in the dental field in clinical practice, to guide bone or periodontal tissue regeneration and serve as a protective barrier, preventing gingival connective tissue from coming into contact with the tooth root surface [100].

Collagen was the most researched substance when absorbable membranes, primarily made of polymeric materials, were first successfully used in the early 1990s. In addition to avoiding the need for additional surgery, absorbable collagen membranes can hasten the healing of soft tissues and effectively eliminate bacterial contamination and self-limiting infection when they become exposed. Inductive tissue regeneration can be accomplished by using a number of techniques involving absorbable membranes, such as electrospinning and freeze-drying [101].

**Figure 5:**
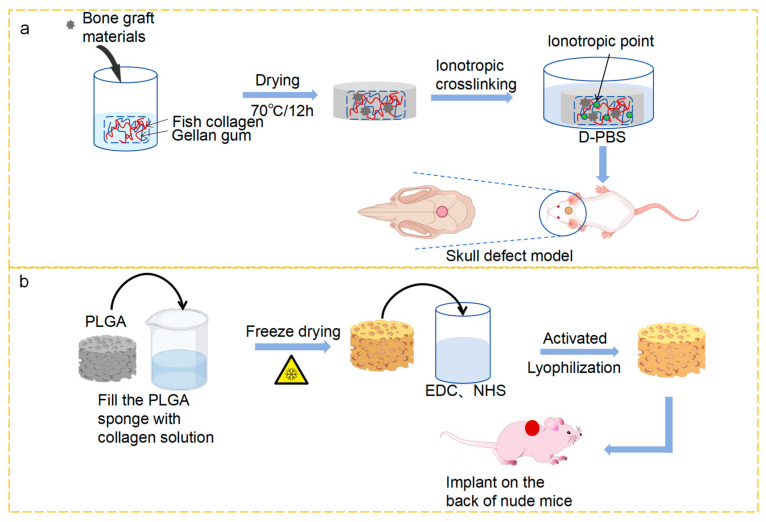
(**a**) Schematic illustration for bone regeneration in vivo using a biomedical membrane made of fish collagen/gellan gum containing bone graft materials [69]. (**b**) Schematic diagram of intracorporeal implantation of collagen sponge made using a porous sponge template of poly (lactic acid-co-glycolic acid) (PLGA) and collagen solution for cartilage regeneration [103].

The fabrication of collagen membranes to direct bone regrowth using materials for bone grafting, which are mostly employed in defect areas to stimulate bone regeneration, has recently attracted much attention. The technology used in orthopedic treatment has rapidly improved in the past few decades, and the development and application of collagen membranes derived from marine sources have increased significantly. Bahrizadeh et al. [102] reported that collagen membranes made from fish swim bladders had a more significant and economical osteogenic effect on the rat cranial skull than bovine-derived collagen membranes. It can be seen from the above study that the physicochemical properties, biological activity, and performance of MC in cell culture in vitro have undergone significant changes. However, the results of this study cannot be well applied in the clinic due to the inherent limitations of collagen materials, so the physicochemical properties of collagen can be greatly enhanced by combining MC with other materials for modification, making collagen more suitable for the fabrication of collagen membranes that can repair bones. Kim et al. [69] used gellan gum and collagen extracted from fish skin to prepare GBR membranes loaded with bone graft material for guided bone regeneration therapy (Figure 5a). Jin et al. [48] added nanohydroxyapatite to fish collagen with low immunogenicity, and nanofiber membranes prepared by electrospinning had an appropriate degradation rate, had good biocompatibility, met the biosafety evaluation requirements, and showed the potential of excellence in guiding bone or tissue regeneration.

### 4.4. Collagen Sponges

Collagen sponges are among the most valuable biomaterials, attributed to their remarkable functions and properties that render them amenable to treatment, sterilization, and preservation [104]. Collagen sponges can activate bone-forming proteins in tissues, which support the synthesis of new bone. These proteins are rapidly degraded and absorbed in the human body and are widely used as highly porous and open-structured three-dimensional biomaterials in biomedical engineering [105]. The most common method for preparing collagen sponges is freeze-drying; under regulated conditions, the freeze-drying process produces high-quality, shelf-stable implants. In addition, freeze-drying prevents the removal of the blocking solvent in the event that the collagen is chemically altered due to conditions such as high temperature and pressure [106].

It has been reported in the literature [107] that recombinant human BMP combined with an absorbable collagen sponge has a good osteogenic effect on skull defects in rats [108,109]. The FDA has approved the use of rhBMP-2 and rhBMP-7 in combination with collagen sponges for tibial nonunion, oral and maxillofacial reconstruction, and spinal fusion [110].

A modified collagen sponge is more conducive to cell culture and proliferation. Zheng et al. [103] were able to achieve gradual proliferation and cartilage differentiation by binding collagen sponges and collagen hydrogels to inducible factors. The incorporation of sponge collagen with inducible factors can serve as an effective platform for cartilage tissue engineering and stem-cell-induced chondrogenic differentiation (Figure 5b). Zhang et al. [111] constructed BM-MSC–collagen sponges that were combined with TGF-β1 through cyclic mechanical stretching, which synergistically promoted cell differentiation into tendon cells and enhanced tendon regeneration.

## 5. Future and Challenge

### 5.1. Safety

Despite its numerous advantages, MC also exhibits certain limitations as a biomedical material. One of the primary concerns is its potential immunogenicity. Although MC generally has low immunogenicity, there remains a possibility that certain populations may elicit an immune response [112]. This potential risk necessitates thorough evaluation in clinical applications, particularly for long-term implanted bone repair materials. An immune response could lead to inflammation, tissue rejection, and other adverse effects, thereby compromising the efficacy and safety of bone repair [8]. Consequently, during the extraction and preparation of MC, it is imperative to employ advanced purification techniques to eliminate impurities and antigenic components that may trigger immune reactions. Additionally, chemical modification or genetic engineering can be utilized to further mitigate immunogenicity and enhance the safety profile for clinical use [113]. Sterilization represents a significant challenge in the biomedical applications of MC biomaterials, as conventional methods, including heating, high pressure, and radiation sterilization, may compromise their performance and biological activity to varying degrees [114]. This necessitates the investigation of advanced technologies, such as photodynamic sterilization, which are designed to maintain sterility while preserving the structural and functional integrity of these materials, thereby enhancing the clinical safety of marine-collagen-based biomaterials [115].

### 5.2. Usability

Although MC can provide suitable mechanical properties for various applications, it does not always meet the specific requirements of certain tissues or implants. This is particularly true in the context of bone repair materials, where strength requirements are more stringent [116]. In response to this challenge, researchers are investigating methods to enhance the mechanical properties of collagen by modifying its structure and incorporating additional materials [116]. By strategically designing the composition and architecture of composite materials and leveraging the advantages of different components, it is possible to improve overall mechanical properties to fulfill the demands for mechanical support in bone repair [117].

The synchronization of the MC degradation rate with the growth rate of new bone is crucial. If the degradation occurs too rapidly, it may fail to provide adequate support and guidance for new bone formation. Conversely, if the degradation is too slow, it can impede the remodeling and integration of new bone tissue [54]. Currently, accurately regulating this degradation rate in vivo remains a challenge, as variations in individual physiological states and metabolic levels add complexity to regulatory efforts [118]. Future research will concentrate on cross-linking, chemical modification, and other innovative technologies aimed at enhancing stability while precisely controlling the degradation rate and improving the mechanical properties of materials.

### 5.3. Standardization

Despite the extensive applications of MC biomaterials in the medical field, certain areas may still be the focus of further research. The extraction and preparation procedures of marine collagen are intricate. Different extraction methods entail diverse process conditions, leading to significant variations in product quality. Owing to the absence of unified quality standards and specifications, clinical applications are impeded [119]. In the large-scale production of products with specific performance requirements, high cost and complex operation limit the application of MC in bone repair materials. Consequently, the development of an efficient and cost-effective extraction and preparation process is a pivotal step [15]. Establishing unified and standardized quality standards and testing methods, strictly controlling production indicators, and ensuring stable and consistent product quality are of utmost importance for future clinical applications.

## 6. Conclusions and Prospects

Marine-derived collagen (type I) has proved to significantly improve bone tissue regeneration. MC has good biological properties, reduces the rejection of foreign substances, avoids concerns about religious beliefs and potential risks, and can be used to repair almost all types of bone tissue within the human body.

The utilization of MC has several benefits, including its degradability, low immunogenicity, strong biocompatibility, and safety, which gives it multiple application prospects in the domain of BTE. The mechanical and physicochemical properties of collagen must vary according to different tissue repair types. By modifying the chemical structure of collagen, one can enhance both its biological and physicochemical characteristics, improving its applicability for a range of bone tissue restoration techniques. Collagen is commonly modified or mixed with other substances to create a range of bone replacement materials, including collagen hydrogels, scaffolds, membranes, and sponges, for use in bone grafting applications. Collagen material delivers growth factors, cells, medications, and genes by combining with materials with better osteoconductivity, osteoinductivity, and osteogenic capacity, making them more suitable for bone tissue repair.

MC is a potential alternative resource. Because MC is a plentiful and easily extracted byproduct, less fishery waste is produced, and the low-value byproduct has greater economic value. The exploitation of marine organisms can have a negative impact on populations and may have varying degrees of impact on ecosystems as well as on fishery resources, potentially depleting the latter. The large-scale exploitation of marine organisms with low reproductive capacity may be restricted in the context of sustainable development. Therefore, when utilizing marine animals for biomedical tissue engineering applications, it is essential to consider the preservation of aquatic biological resources and the source of commercial raw materials for MC.

We are currently in the early stages of researching the application of modified MC in tissue engineering. Further investigations are needed in this area. We should employ various methodologies to examine and evaluate the production of scaffold materials and alternative MC types, as well as their mechanical attributes, pore size features, swelling, and rates of degradation, such as in the cases of skin, cartilage, and bone restoration. In light of the possibility of allergies to some marine species, it is crucial to assess the safety of clinical trials and efficacy in real-world applications. A novel concept for the therapeutic use of bone tissue healing in the next ten years is the synthesis of functional collagen materials from the ocean in conjunction with bone regeneration.

## Figures and Tables

**Table 1:** Application of marine collagen in various bone tissues.

Collagen Source	Binding Materials	Cross-Linking Agent	Mechanical Behavior	Forms of Action	Site of Action	Action Cells	Example
Swim bladder Bester sturgeon fish	HAP	GA, genipin, and N,N′-methylenebis (acrylamide)	Increased modulus, similar to articular cartilage	Hydrogel	Cartilage of the knee bones		[62]
Scale		Epigallocatechin gallate	Increased modulus of elasticity and reduced degradation rate	GBR membrane	Skull	Sprague Dawley rat bone marrow mesenchymal stem cells	[63]
Stingray skin	Oyster shell powder	Genipin	Improved mechanical properties	Composite bracket		MC3T3-E1 cells	[64]
Jellyfish	Fucose (derived from brown algae) and chitosan (from the squid barrier)	Natural cross-linking (polyelectrolyte interaction)	Increased viscosity and adhesion values	Hydrogel			[65]
Medical-grade fish skin	PLA-glycolic acid	Conjugated electrospinning technique		Nanofiber membrane		Mouse fibroblasts, L929	[66]
Cod skin	Silica-based materials	EDC	Increased compression modulus and improved swelling properties	3D composite stent		L929 fibroblasts	[67]
Salmon skin	Femoral condyles	EDC	Improved mechanical properties	Fibril gel			[68]
Fish	Gellan gum	D-PBS	Increased tensile strength	GBR membrane	Skull	L292 cells	[69]
Salmon	Expanded polytetrafluoroethylene			Periosteal sheet	Alveolar bone		[70]
Bluefin tuna	Blue crab chitosan		Enhanced stability, strength, and viscoelasticity	Hydrogel			[71]
Jellyfish	Hydroxyphenylpropionic acid	Horseradish peroxidase, hydrogen-peroxide-catalyzed oxidative coupling (enzymatic cross-linking)	Stiffness improvement	Hydrogel	Nasal septal cartilage	Chondrocytes	[72]
*Macruronus novazealandii* skin	Methacrylates	Ultraviolet (physical cross-linking)	Proper viscosity and shear thinning	3D-printed bioink hydrogel		L929 fibroblasts	[73]
Nile *tilapia* skin	HAP	Heat cross-linking	Superior strength, higher flexibility, elasticity, and heat resistance	GBR membrane		MC3T3-E1 cells	[74]
Scale	Oxidized chondroitin sulfate, polyethylene glycol acrylate (PEGDA)	Ammonium persulfate, TEMED	Increased compressive strength	Double-layer hydrogel stent	Articular cartilage	ADMSCs	[75]
*Paralichthys olivaceus* skin	Polycaprolactone, alginate	EDC	Higher water absorption capacity	3D scaffold	Femur area		[76]
Jellyfish and blue shark skin	Chitosan, fucoidan	Natural cross-linking	Increased elastic–solid properties and mechanical stability	Hydrogel	Articular cartilage tissue	ATDC5	[77]
Blue shark skin	BAp	EDC	Higher compressive modulus	Composite scaffolds	Distal lateral part of femoral condyle		[78]
Jellyfish	Mineralized salmon collagen that mimics biology	EDC		Duplex brackets	Osteochondral tissue	Human mesenchymal stromal cells	[79]
Osteogenic abalone	Poly(ε-caprolactone).	EDC, NHS	Decreased stress rate and increased strain rate	3D bracket	Shin	Mouse mesenchymal stem cells	[80]
Shark skin	Apatite, a marine mineral derived from shark teeth	EDC/NHS or HMDI	Increased ductility	3D bracket	Hard tissue	Osteoblast-like cell lines	[81]
Fish scales	Chitosan, HAP, and β-tricalcium phosphate	GA	Reduced degradation rate and improved swelling properties	Composite stents	Osteoporosis	NIH/3T3, MG-63	[82]
Sponge	Chitosan, HAP	Natural cross-linking	Decreased water absorption	Composite stents		Osteoblast-like MG-63 cells	[83]
Skin of alfalfa	HAP carbonate, polycaprolactone		Increased modulus of elasticity	3D printed scaffold	Skull	MC3T3-E1	[84]
Fish	Alginate, polycaprolactone, mesenchymal tannins solution	EDC		Composite stents	Thigh bone	MG63 cells	[85]
Jellyfish		EDC	Higher thermal stability	Bracket	Cartilage tissue of bovine joints	Bovine cartilage protein cells	[86]
Jellyfish	Alginate	EDC	High elasticity	Bracket	Cartilage	hMSC	[87]

## Data Availability

Not applicable.
